# The Extracts and Major Compounds Derived from Astragali Radix Alter Mitochondrial Bioenergetics in Cultured Cardiomyocytes: Comparison of Various Polar Solvents and Compounds

**DOI:** 10.3390/ijms19061574

**Published:** 2018-05-25

**Authors:** Yun Huang, Kenneth Kin Leung Kwan, Ka Wing Leung, Huaiyou Wang, Xiang Peng Kong, Tina Ting Xia Dong, Karl Wah Keung Tsim

**Affiliations:** 1 Shenzhen Key Laboratory of Edible and Medicinal Bioresources, Shenzhen Research Institute, Hi-Tech Park, Nanshan, Shenzhen 518000, China; yhuangbr@connect.ust.hk (Y.H.); lkwing@ust.hk (K.W.L.); hyw@ust.hk (H.W.); xpkong@ust.hk (X.P.K.); botina@ust.hk (T.T.X.D.); 2 Division of Life Science and Center for Chinese Medicine, The Hong Kong University of Science and Technology, Clear Water Bay, Hong Kong, China; klkwan@connect.ust.hk

**Keywords:** Astragali Radix, astragaloside IV, genistein, mitochondrial bioenergetics, oxygen consumption rate

## Abstract

Astragali Radix (AR) is a widely used “Qi-invigorating” herb in China for its tonic effects in strengthening biological tissues. The extract of AR contains abundant antioxidants, including astragalosides and isoflavonoids. However, very few reports have systematically measured the effects of the major components of AR on cell mitochondrial bioenergetics. Here, a systemic approach employing an extracellular flux analyzer was developed to evaluate mitochondrial respiration in cultured cardiomyocyte cells H9C2. The effects of different polar extractives, as well as of the major compounds of AR, were compared. The contents of astragaloside IV, calycosin, formononetin, and genistein in the AR extracts obtained by using water, 50% ethanol, and 90% ethanol were measured by liquid chromatograph-mass spectrometer (LC–MS). The antioxidant activities of the AR extracts, as well as of their major compounds, were determined by measuring the free radical scavenging activity and protective effects in *tert*-butyl hydroperoxide (tBHP)-treated H9C2 cells. By monitoring the real-time oxygen consumption rate (OCR) in tBHP-treated cardiomyocytes with a Seahorse extracellular flux analyzer, the tonic effects of the AR extracts and of their main compounds on mitochondrial bioenergetics were evaluated. AR water extracts possessed the strongest antioxidant activity and protective effects in cardiomyocytes exposed to oxidative stress. The protection was proposed to be mediated via increasing the spare respiratory capacity and mitochondrial ATP production in the stressed cells. The major compounds of AR, astragaloside IV and genistein, showed opposite effects in regulating mitochondrial bioenergetics. These results demonstrate that highly polar extracts of AR, especially astragaloside-enriched extracts, possess better tonic effects on mitochondrial bioenergetics of cultured cardiomyocytes than extracts with a lower polarity.

## 1. Introduction

Astragali Radix (AR), derived from the dry roots of *Astragalus membranaceus* (Fisch.) Bge. var. *mongholicus* (Bge.) Hsiao and *Astragalus membranaceus* (Fisch.) Bge, is one of the most widely used traditional folk medicines in Asia because of its beneficial effects in invigorating “Qi” and strengthening tissues [1]. According to traditional Chinese medicine (TCM) theory, the interaction between “Yin and Yang” produces “Qi”. AR acts mainly by tonifying defensive Qi, which protects the body against disease-causing factors [2]. Hence, multiple pharmacological effects of AR have been reported, e.g., immuno-modulation [3], anti-aging [4], anti-oxidation [5], and enhancement of cardiovascular function [6].

Being considered as a powerful exogenous source of antioxidants, AR provides obvious protective effects to organs in various models of oxidative stress-related diseases [7,8,9]. Astragaloside and isoflavonoid were proposed to be the major antioxidants of AR [10]. Astragalosides, especially astragaloside IV, have been proved to exert remarkable antioxidant activity and protective effects on mitochondria [11]. Astragaloside IV suppressed heat-induced apoptosis in bronchial epithelial cells by inhibiting the activation of mitochondrial Ca^2+^ uniporter, mitochondrial depolarization, and reactive oxygen species (ROS) production [12]. Other strong antioxidant components of AR are isoflavonoids, e.g., calycosin, formononetin, and genistein. Calycosin showed anti-apoptotic effects by enhancing Akt phosphorylation and activating ERα/β in cardiomyocytes exposed to oxidative stress [13], while formononetin was observed to attenuate apoptosis by regulating ROS formation in various cancer models [14,15]. Genistein was reported to be an anti-cancer agent [16], which could induce the disruption of mitochondrial membrane potential, the release of cytochrome c, and the activation of the apoptosome [17]. Chemical analysis has shown that AR contains different active ingredients, which therefore could account for the aforementioned biological activities [18].

Mitochondria are responsible for the production of ROS in the process of generating ATP. Defects in mitochondria are proposed to be the primary cause of energy decline, as well as of the onset of the aging process [19]. The profile of mitochondrial bioenergetics could be an indicative measurement of a cell energy status. The profile, e.g., the oxygen consumption rate (OCR) and the spare respiratory capacity (SRC), corresponds to the ability of mitochondria to increase ATP. In addition to the spare respiratory capacity, the maximal respiration of cells could be affected by the basal respiration consisting in proton leak and conventional mitochondrial ATP production. Proton leak from the mitochondrial inner membrane results in the uncoupling of oxidative phosphorylation, which is cytoprotective [20]. In addition, this leakage could downregulate ROS generation [21]. However, the proton leak also represents basal respiration not coupled to mitochondrial ATP production, and this could be considered as a sign of mitochondrial damage [22].

AR is considered a “Qi”-invigorating herb, which suggests its beneficial effect resulting in the production of ATP. The possible functions of AR in mitochondrial protection and elimination of ROS formation have been reported [23,24]. However, the real-time effect of AR in mitochondrial bioenergetics of living cells has rarely been studied. Here, a systematic approach to evaluate mitochondrial bioenergetics of intact cultured cardiomyocytes was adopted by using an extracellular flux analyzer. The major compounds of AR, e.g., astragaloside IV, calycosin, formononetin, and genistein, were also analyzed, and their functions in mitochondria were compared.

## 2. Results

### 2.1. Quantification of Chemicals and Total Phenolic Compounds in Different Extracts

**Figure 1:**
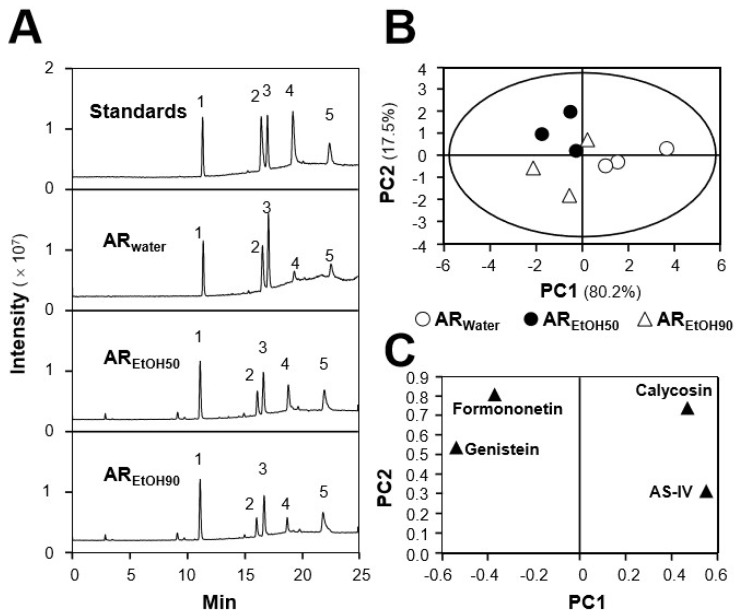
Principal component analysis (PCA) of major compunds in different Astragali Radix (AR) extracts. (**A**) The identification of ginsenoside Rg1 (1; internal control marker), calycosin (2), astragaloside IV (3), genistein (4), and formononetin (5) was made by an MS detector. Representative chromatograms of chemical markers (Standards), water extract of AR (AR_water_), 50% ethanol extract of AR (AR_EtOH50_), and 90% ethanol extract of AR (AR_EtOH90_) under MRM mode are shown. (**B**) The scoring plot of different AR extracts is presented by comparing the contents of chosen major compounds. PC1 and PC2 described ~80.2% and ~17.5% of total variability, respectively. (**C**) The loading plot of PC1 versus PC2 for four compounds is shown. *n* = 3.

The contents of calycosin, astragaloside IV, genistein, and formononetin in a water extract of AR (AR_water_), a 50% ethanol extract of AR (AR_EtOH50_), and a 90% ethanol extract of AR (AR_EtOH90_) of three different batches of AR were quantified by LC–MS in multiple reaction monitoring (MRM) detection mode (Table 1 and Figure 1A). The analytical method was optimized on the basis of previous reports [25]. As summarized in Table S1, the fragmentor energy, collision energy, and ion pairs were optimized to attain the highest abundance of detected chemicals. In MS/MS analysis, the calibration curves of the four markers were linear over a concentration range of 1–1000 ng/mL, and the *r*^2^ values of these calibration curves were higher than 0.990 in the analysis (Table S2). The investigation of precision, repeatability, and recovery of those four chemicals in AR extracts proved that the established method was robust enough for simultaneous quantification of various chemicals in AR, as summarized in Table S3.

Representative chromatograms of standard markers (Standards), AR_water_, AR_EtOH50_, and AR_EtOH90_ under MRM mode are shown (Figure 1A). The chemical markers of ginsenoside Rg1 (internal control), calycosin, astragaloside IV, genistein, and formononetin are indicated. After determination of the contents of selected markers in three batches of AR, a principal component analysis (PCA) analysis was conducted to differentiate the samples extracted with water from those extracted with different ethanol aqueous solutions. As shown in Figure 1B, the two ranking PCs (PC1 and PC2) accounted for 97.7% of total variance, and AR extracts could be obviously distinguished into three distinct groups, according to the extracting solvents. The role of each variable (calycosin, astragaloside IV, genistein, and formononetin) in discriminating the extracts is shown in the loading plot (Figure 1C). Combined with the contents of selected markers recorded in Table 1, the contents of calycosin and astragaloside IV in AR_water_ extract were generally higher than those in AR_EtOH50_ and AR_EtOH90_, while the for other two compounds the results appeared reversed. The contents of these markers could serve as quality control parameters.

**Figure 2:**
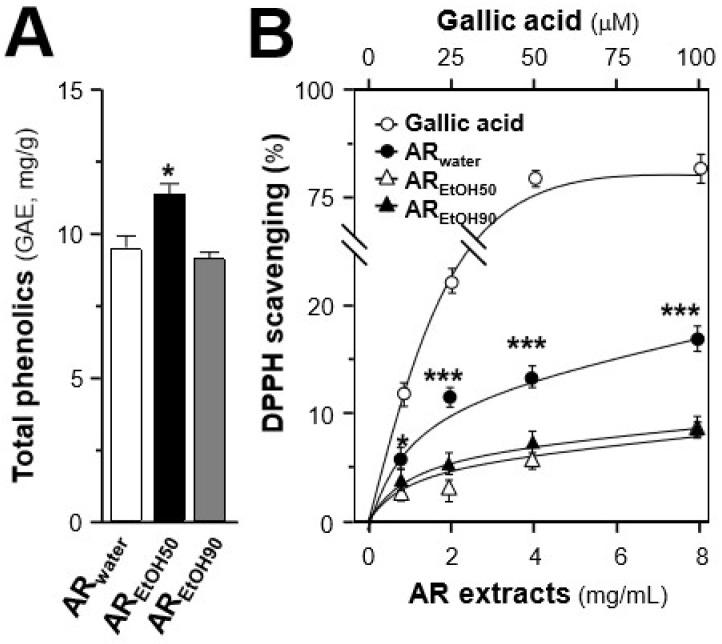
Comparison of total phenolic contents and 1,1-Diphenyl-2-picrylhydrazyl radical 2,2-Diphenyl-1-(2,4,6-trinitrophenyl)hydrazyl (DPPH) radical scavenging activity of different AR extracts. (**A**) The total phenolic contents of the AR extracts was determined using the Folin–Ciocalteu assay. Gallic acid was used as a reference compound, and the total phenolic content of each extract was expressed as the value of gallic acid equivalent (i.e., GAE in mg/g). (**B**) The antioxidant effects of the AR extracts were determined using the DPPH radical scavenging assay. Gallic acid was used as a positive control. All data are expressed as mean ± SD, *n* = 5. Statistical comparison was made with the sample with the lowest value of the corresponding concentration, * *p* < 0.05, *** *p* < 0.001.

Apart from the quantification of bioactive markers, the antioxidant effects of the AR extracts, i.e., AR_water_, AR_EtOH50_ and AR_EtOH90_, were evaluated by measuring their total phenolic content and free radical scavenging activity. Different extracts of AR from batch 1 were used for the subsequent analyses. The total phenolic contents of AR_water_, AR_EtOH50_, and AR_EtOH90_ were determined in reference to gallic acid (Figure 2A). Among the three extracts, AR_EtOH50_ exhibited a significant higher content of phenolic compounds, equivalent to ~11.7 mg gallic acid/g of sample, while AR_water_ and AR_EtOH90_ showed ~9.4 mg gallic acid/g and ~9.1 mg gallic acid/g, respectively. Inconsistent with this result, AR_water_ showed a significant higher free radical scavenging activity than the ethanol extracts (Figure 2B), indicating that the phenolic compounds might not be the only antioxidant present in AR.

### 2.2. Protection Effects of the AR Extracts in H9C2 Cells Subjected to Oxidative Stressed

**Figure 3:**
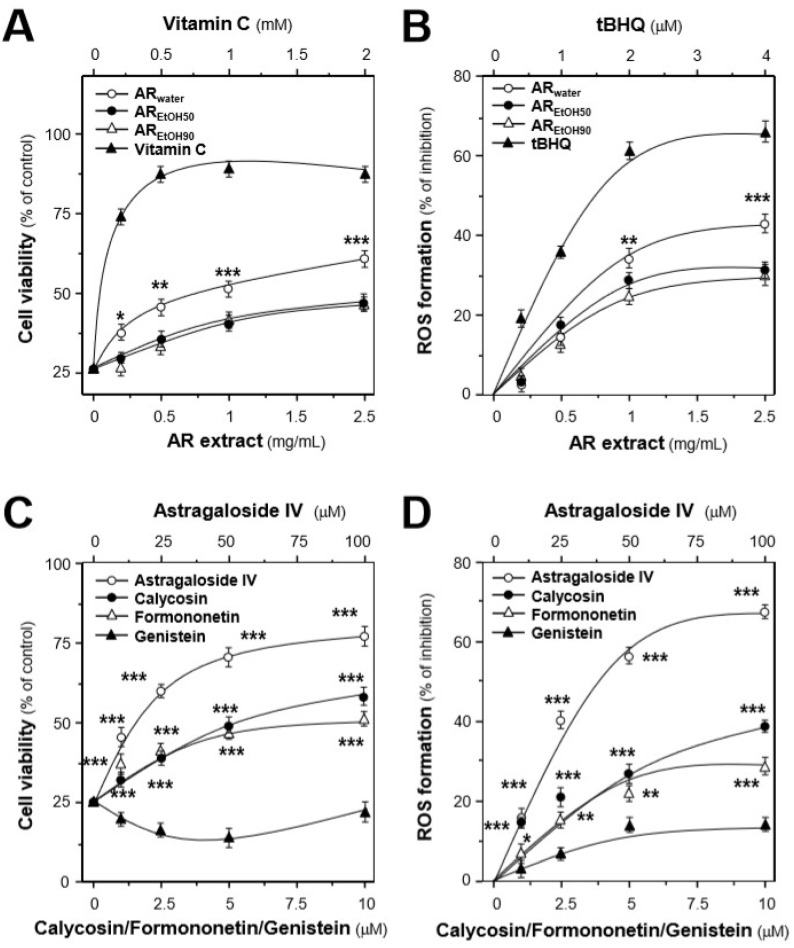
Protection effects of the AR extracts and major compounds in H9C2 cells against oxidative stress. (**A**) The dose-dependent response was determined by pre-treating the cultures with AR extracts (batch AR-1), i.e., water extract of AR (AR_water_), 50% ethanol extract of AR (AR_EtOH50_), and 90% ethanol extract of AR (AR_EtOH90_) for 24 h before the addition of tBHP (150 μM). Vitamin C at various concentrations served as a positive control. (**B**) Cultured H9C2 cells were pre-treated with the AR extracts or *tert*-butyl hydroquinone (tBHQ) for 24 h and then exposed to tBHP (150 μM) for 1 h. The result is in percentage of ROS formation relative to the tBHP-treated control. (**C**) Cultured H9C2 cells were treated with the AR major compounds for 24 h before the addition of tBHP (150 μM). Then, the cell viability was recorded. (**D**) The effects of the chosen markers on ROS formation were compared in tBHP-treated cardiomyocytes. All data are expressed as mean ± SD, *n* = 5, each with triplicate samples. Statistical comparison was made with the sample with the lowest value of the corresponding concentration, * *p* < 0.05, ** *p* < 0.01, *** *p* < 0.001.

H9C2 cell, an embryonic cardiomyocyte cell line, was selected here because of its robust and fast reaction to various stimuli. By treating with different concentrations of tBHP, a stress inducer, cell death of H9C2 cells and intracellular ROS level were induced in a dose-dependent manner. tBHP at 150 μM caused the maximal induction of ROS (Figure S1), and this concentration was used for the subsequent experiments [26]. By using a cell viability assay, the concentration range of the tested extracts and markers was also optimized (Figure S2). The results revealed that all AR extracts could dose-dependently protect the cells against oxidative insult (Figure 3A). Among the three extracts, AR_water_ possessed the best protective effects up to over 60% as compared with the control, while AR_EtOH50_ and AR_EtOH90_ showed almost identical effects corresponding to ~40% compared to the control. Consistent with the protection effects, the AR extracts could dose-dependently decrease tBHP-induced ROS formation: AR_water_ showed the greatest inhibitory effect corresponding to ~42%, as compared with those of the control and of the other extracts (Figure 3B).

The protective effects of the four chemical markers in stressed cells were also evaluated. Apart from genistein, the three compounds could dose-dependently increase cell survival (Figure 3C). Astragaloside IV showed the best protective effects of ~76%, compared with calycosin (~58%) and formononetin (~51%). Genistein showed slight inhibitory effects on tBHP-induced ROS formation. Among the tested markers, astragaloside IV possessed the best inhibitory effect of ~68%, which was consistent with the enhancement of cell viability (Figure 3D).

**Figure 4:**
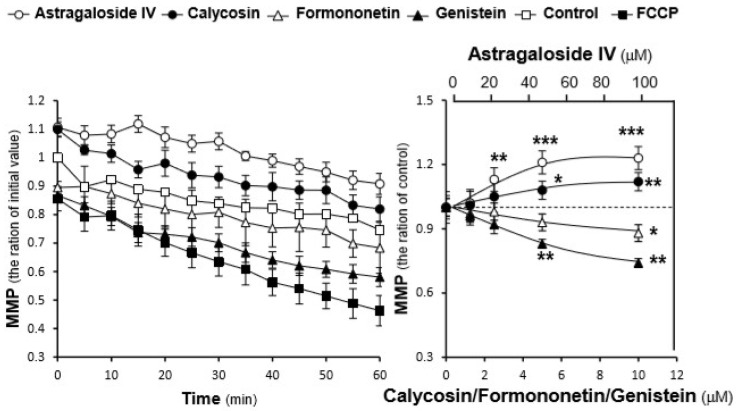
AR compounds regulate the mitochondrial membrane potential of H9C2 cells. Pretreated in 96-well black multi-well plates with a clear bottom for 24 h, H9C2 cells were stained with JC-1 and then washed with PBS-A twice. After adding the indicated concentration of compounds in the wells, the fluorescence of JC-1 aggregates in each sample was measured every 5 min for 60 min at 37 °C. Here, carbonyl cyanide-p trifluoromethoxyphenylhydrazone (FCCP, 100 μM) was used as the positive control. Time course of markers-induced changes in the mitochondrial membrane potential in H9C2 cells (**left panel**). The data are expressed as the ratio of fluorescence intensity of JC-1 aggregates to the respective initial (i.e., 0 min) value. The values given are mean ± SD, with *n* = 3. The dose-dependent effects of the chosen markers on the mitochondrial membrane potential were recorded (**right panel**). The data are expressed as the ratio of fluorescence intensity of JC-1 aggregates to the corresponding value of the control at 60 min. The values given are mean ± SD, with *n* = 3. Statistical comparison was made with the control, * *p* < 0.05, ** *p* < 0.01, *** *p* < 0.001.

To further investigate the effects of AR chemicals on mitochondrial integrity, the mitochondrial membrane potential was measured. As shown in Figure 4, H9C2 cells incubated with astragaloside IV showed a significant slower fluorescence decay rate, as compared with the control during the first 20 min, resulting in a mitochondrial membrane potential ~1.3-fold higher at the end of the recording. Similar to astragaloside IV, calycosin also showed a higher mitochondrial membrane potential throughout the observation process. However, formononetin and genistein both gradually and significantly decreased the mitochondrial membrane potential in H9C2 cells. Furthermore, the concentration-dependent effects of these compounds on the mitochondrial membrane potential were also revealed (Figure 4). By comparing with the mitochondrial membrane potential of the control at 60 min, astragaloside IV and calycosin could dose-dependently increase the mitochondrial membrane potential in cultured H9C2 cells, while formononetin and genistein showed a downward trend.

### 2.3. Effects of the AR Extracts and Compounds on Mitochondrial Bioenergetics

**Figure 5:**
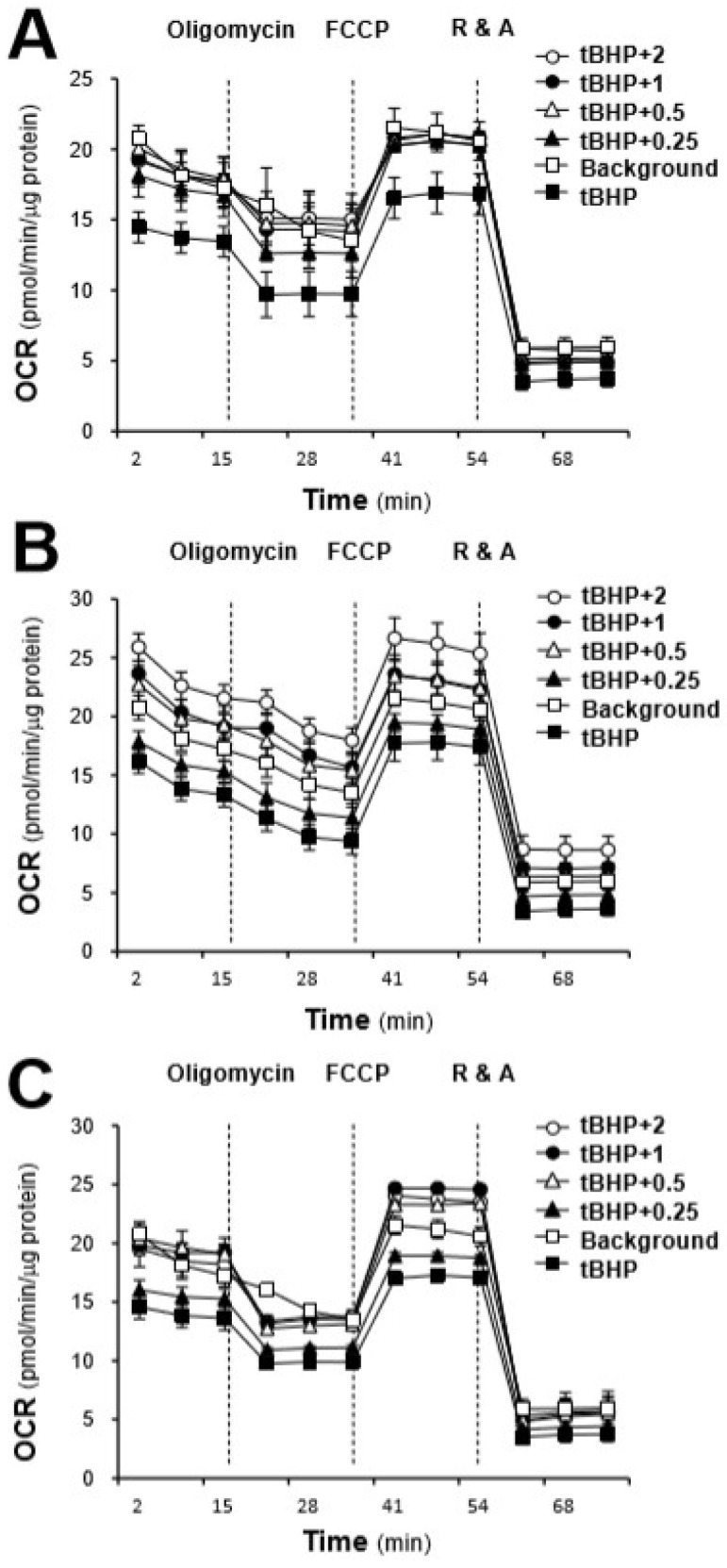
The AR extracts modulate the mitochondrial bioenergetics of H9C2 cells. Cultured H9C2 cells were treated with the AR extracts (from 0.25 to 2 mg/mL as indicated) for 24 h before measuring the oxygen consumption rate (OCR) with the XFp Cell Mito Stress Test. The responses of H9C2 cells after oligomycin (1 μM), FCCP (3 μM), and rotenone/antimycin A (R&A at 1 μM) treatments were recorded. The dotted lines denote the times at which the three inhibitors were applied. Effects of various extracts of AR: (**A**) water extract of AR, (**B**) 50% ethanol extract of AR, (**C**) 90% ethanol extract of AR, at various concentrations on the OCR for four respiration states of H9C2 cells are shown. The OCR values were normalized with respect to the cellular protein content/well. The background are the untreated cell. The data are expressed as mean ± SD, *n* = 3; each experiment was performed with triplicate samples.

**Figure 6:**
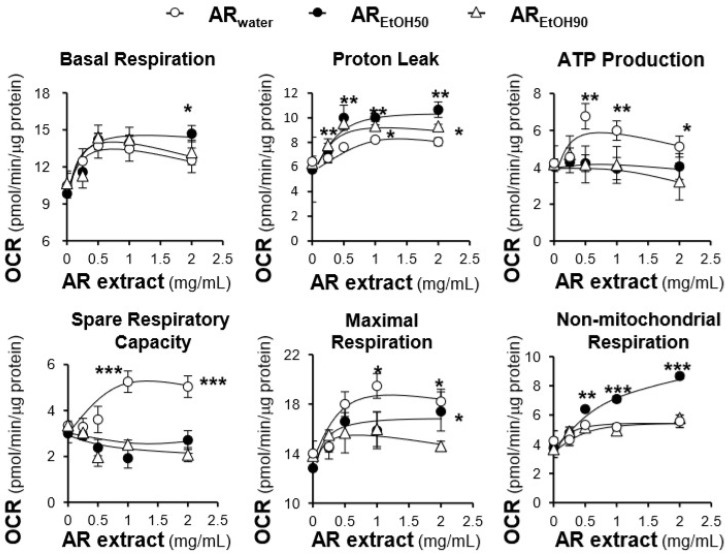
Various parameters of mitochondrial respiration in AR-treated H9C2 cells are compared. Cultured H9C2 cells were treated as in Figure 5. The effects of increasing concentration of AR extracts on basal respiration, proton leak, mitochondrial ATP production, spare respiratory capacity, maximal respiration, non-mitochondrial respiration were measured and compared amongst the three AR extracts. The OCR values were normalized with respect to the cellular protein content. The data are expressed as mean ± SD, *n* = 3, each with triplicate samples. Statistical comparison was made with the sample with the lowest value of the corresponding concentration, * *p* < 0.05, ** *p* < 0.01, *** *p* < 0.001.

By using a Seahorse extracellular flux analyzer, the effects of the AR extracts, i.e., AR_water_, AR_EtOH50_ and AR_EtOH90_, on the OCR of tBHP-treated H9C2 cells were plotted against time. The profile of the OCR in tBHP-treated H9C2 cells was obtained and appeared to be altered by treatments with the various AR extracts (Figure 5). According to the conversion relationship (Figure S3) [26], various parameters of mitochondrial bioenergetics, i.e., basal respiration, proton leak, mitochondrial ATP production, spare respiratory capacity, maximal respiration, and non-mitochondrial respiration, were evaluated (Figure 6). All AR extracts could dose-dependently increase basal respiration, proton leak, maximal respiration, and non-mitochondrial respiration to different degrees (Figure 6). Among these responses, AR_EtOH50_ induced the greatest increase in basal respiration, proton leak, and non-mitochondrial respiration; the increase was from 1.6 to 2 folds at the maximum concentration. AR_water_ only increased basal respiration, proton leak, and non-mitochondrial respiration from 1.2 to 1.3 folds, which was significantly lower than compared to AR_EtOH50_. Similarly, the effects of AR_water_ were totally different from those of AR_EtOH50_ and AR_EtOH90_ with respect to mitochondrial ATP production and spare respiratory capacity. AR_water_ could dose-dependently increase mitochondrial ATP production and spare respiratory capacity up to ~1.5 folds, while AR_EtOH50_ and AR_EtOH90_ showed no difference. Although AR_water_ and AR_EtOH50_ showed opposite effects on mitochondrial ATP production, spare respiratory capacity, and proton leak, both extracts showed a considerable induction of maximal respiration, which was significantly higher than that observed for AR_EtOH90_.

**Figure 7:**
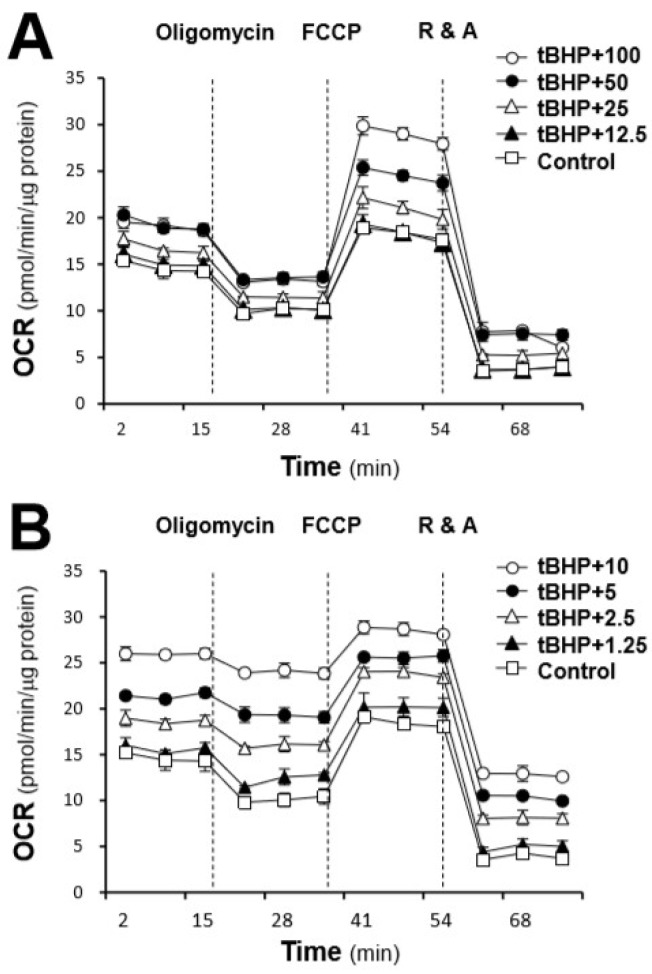
Effects of the major compounds of AR, i.e., (**A**) astragaloside IV and (**B**) genistein, on tBHP-treated H9C2 cells against oxidative stress. Cultured H9C2 cells were pre-treated with astragaloside IV (0–100 μM) and genistein (0–10 μM) for 24 h before being exposed to tBHP (30 μM) for 1 h. The OCR values were normalized with respect to the cellular protein content. The control was tBHP-treated cultures. The data are expressed as mean ± SD, *n* = 3, each with triplicate samples.

**Figure 8:**
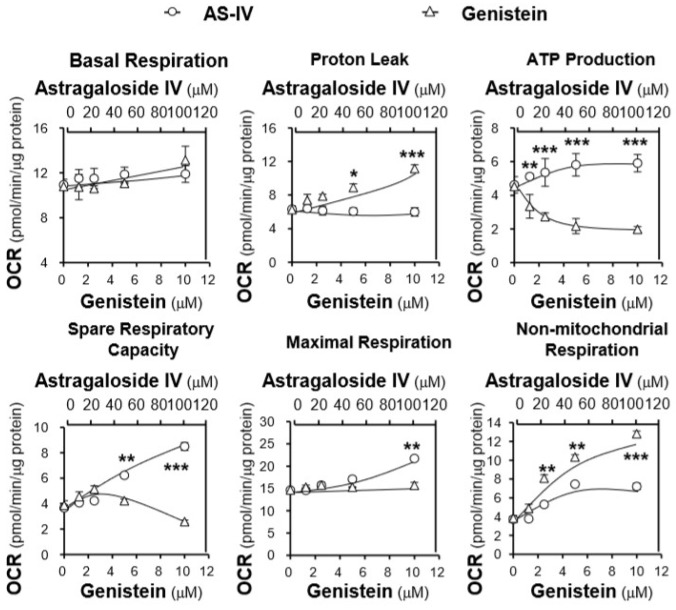
Mitochondrial respiration in tBHP-treated H9C2 cells after pre-treatment with astragaloside IV and genistein. Cultured H9C2 cells were treated as in Figure 7. The basal respiration, proton leak, mitochondrial ATP production, spare respiratory capacity, maximal respiration, non-mitochondrial respiration were measured. The OCR values were normalized with respect to the cellular protein content/well by the Bradford’s method. The data are expressed as mean ± SD, *n* = 3, each with triplicate samples. Statistical comparison was made with the sample with the lowest value of the corresponding concentration, * *p* < 0.05, ** *p* < 0.01, *** *p* < 0.001.

Furthermore, the effects of the major compounds of AR on the stressed cells were also determined. On the basis of their effects on the mitochondrial membrane potential, astragaloside IV and genistein, representing two different responses, were selected for the determination of their effects on mitochondrial bioenergetics. Compared with the AR extracts, astragaloside IV and genistein altered the OCR profile of tBHP-treated H9C2 cells dose-dependently and more robustly than the AR extracts (Figure 7). However, their effects on these specific parameters were totally different. Pre-treatment with astragaloside IV at 100 μM of tBHP-treated H9C2 cells increased mitochondrial ATP production of ~1.5 folds and the spare respiratory capacity of ~2 folds, compared with the control, while it showed a negligible impact on proton leak, resulting in a slight increase in basal respiration and a significant increase in maximal respiration (Figure 8). In contrast, genistein at 10 μM increased the proton leak up to ~1.7 folds and decreased mitochondrial ATP production to ~50% and the spare respiratory capacity to ~60%, as compared with the control, which resulted in a slight increase in basal respiration and maximal respiration (Figure 8). Moreover, both compounds could dose-dependently increase the non-mitochondrial respiration to different degrees.

## 3. Discussion

AR is well known for its “Qi-invigorating” action in Chinse medicine and is classified as a top-grade herb in *Shen Nong Ben Cao Jing* [10]. Being the major active compounds of AR, astragalosides and isoflavonoids are generally used as standard chemicals for quality control of AR. Here, simultaneous quantification of four major compounds, i.e., calycosin, astragaloside IV, formononetin, and genistein, in three different polar extracts of AR were performed with LC–MS. The contents of astragaloside IV and calycosin in a water extract of AR were higher than in ethanol extracts, which might be due to the highly polar structures of these compounds [27]. Oppositely, formononetin and genistein were present in higher amounts in the ethanol extracts, especially in the 50% ethanol extract [28]. On the basis of the different content of these compounds, different polar extracts could be obviously classified by PCA, and the distribution of the extracts in the PCA scoring plot was highly consistent with the amounts of the major compounds in the loading plot. These results revealed that the chemical composition of AR extracts might differ a lot when different polar solvents are used for AR extraction.

The total phenolic compound content and free radical scavenging activity of the extracts were determined, to have a rough estimation of the extracts’ antioxidant activity. However, the extracts with the most abundant phenolic compounds did not show the best free radical scavenging activity, suggesting that different phenolic compounds might have greatly different antioxidant activity. Thus, the protective effects of the AR extracts and major compounds in cultured H9C2 cells against tBHP-induced oxidative stress were determined. Consistent with their free radical scavenging activity, the water extract showed the best protection in cells exposed to oxidative stress as a result of its inhibitory effects on tBHP-induced ROS. Among the selected AR compounds, astragaloside IV possessed the best protective effects in cultured cells against ROS formation. Interestingly, although genistein showed no influence on cell viability, it could slightly decrease the amount of intracellular ROS. Thus, genistein effect was proposed to be closely related to its effects on mitochondrial permeability transition pore and uncoupling proteins [29]. These results were further confirmed by the analysis of the mitochondrial membrane potential, which was established by determining the different proton concentrations inside and outside the mitochondrial inner membrane [30].

Because of its central role in energy homeostasis, metabolism, signaling, and apoptosis, research on mitochondria is enduring [31]. However, the previous experimental approaches to study mitochondria bioenergetics mainly considered isolated mitochondria, which could result in increased ROS, or other lesions, due to different infiltration environments [32]. Here, the extracellular flux analyzers enabled us to monitor the real-time mitochondrial respiration in live cells, greatly enhancing the credibility of our results [33]. The application of this technology to the AR extracts or to their components is still very limited. Here, only the water extracts of AR showed a dose-dependent enhancement of the spare respiratory capacity and mitochondrial ATP production, which might be due to the high content of tonic compounds in these extracts. This hypothesis was supported by measuring the effects of astragaloside IV and genistein on mitochondrial bioenergetics. In 2015, Lu et al. (2015) found that astragaloside IV could significantly increase mitochondrial OCR and ATP production in vascular smooth muscle cells [34]. Later on, Dong et al. (2017) revealed that the enhancement by astragaloside IV of ATP production was triggered by the stimulation of fatty acid α-oxidation and the inhibition of excessive activation of mitochondrial Ca^2+^ uniporter [35]. Consistent with these studies, the current results showed that astragaloside IV could effectively restore the spare respiratory capacity and ATP production in tBHP-treated cardiomyocytes. Different from astragaloside IV, the effects of genistein on ATP production is still difficult to interpret. According to Rasbach et al. (2008), the treatment of genistein upregulated the expression of PGC-1α and ATP synthase β, which resulted in an increase in O_2_ consumption and ATP production in renal proximal tubule cells [36]. However, Zheng et al. (2000) reported a non-competitive inhibitory action of genistein on mitochondrial proton F0F1-ATPase/ATP synthase in rat brain and liver preparations, and, as such, ATP production was reduced [37]. Genistein has been proposed for the treatment of cancer, neurodegeneration, and cardiovascular and endocrine diseases; however, a detail study of genistein-mediated regulation of redox biology and mitochondrial biogenesis is still missing [17]. Here, we showed that genistein possessed small or even negative effects on the spare respiratory capacity and mitochondrial ATP production, while it could dose-dependently increase proton leak. This explained the reduction of intracellular ROS after treatment with genistein. All in all, the opposite effects of compounds like astragaloside IV and genistein on mitochondrial bioenergetics could be the reason for the different effects of the various AR extracts. 

In summary, the present research provides a comprehensive investigation of the antioxidant activity of AR extracts and of their effects on mitochondrial bioenergetics. To explain the difference of the tonic effects between water extracts and ethanol extracts, a further study of the major components, i.e., astragaloside IV and genistein, was conducted. The results revealed that astragaloside IV showed significant tonic effects in cells exposed to oxidative stress by dose-dependently increasing the spare respiratory capacity and mitochondrial ATP production, while genistein mainly induced proton leak.

## 4. Materials and Methods

### 4.1. Chemicals and Preparation of the RA Extracts

High performance liquid chromatography (HPLC)-grade acetonitrile and ethanol were bought from Merck (Darmstadt, Germany). Ultra-pure water was obtained from a Milli-Q purification system (Millipore, Molsheim, France). Formic acid was purchased from Riedel-de Haen International (Honeywell, Hanover, Germany). The chemical standards of calycosin, astragaloside IV, genistein, and formononetin were purchased from National Institute for the Control of Pharmaceutical and Biological Products (Beijing, China). The purity of these chemical markers was over 98%. Three batches of dried raw materials of *Astragali Radix* (AR; root of *A. membranaceus* var. *mongholicus*) were bought from Shanxi Province of China and then authenticated by Tina Dong at The Hong Kong University of Science and Technology (HKUST), according to the established morphological characteristics. The voucher specimens were stored in the Centre for Chinese Medicine R&D at HKUST. The water extracts (AR_water_), 50% ethanol extracts (AR_EtOH50_), and 90% ethanol extracts (AR_EtOH90_) of RA were prepared using a standardized extraction method. Briefly, 4 grams of the powdered sample were refluxed in 100 mL of solvent for two times (each time for 2 h); the supernatants were combined and then dried under vacuum.

### 4.2. Standardization of Herbal Extracts

The measurement of chemicals in AR_water_, AR_EtOH50_, and AR_EtOH90_ was performed in an Agilent HPLC 1200 series system (Agilent, Waldbronn, Germany), which was equipped with a degasser, a binary pump, an auto sampler, a thermostated column compartment, and a diode array detector (DAD). The samples were separated on an Agilent ZORBAX Eclipse XDB-C18 column (1.8 μm i.d., 50 mm × 4.6 mm; Agilent, Waldbronn, Germany) after filtration with a guard column. The mobile phase was composed of 0.1% formic acid in acetonitrile (A) and 0.1% formic acid in water (B), according to the pre-set gradient program: 0–3 min, linear gradient 20.0–30.0% (A); 3–8 min, linear gradient 30.0–50.0% (A); 8–10 min, isocratic gradient 50.0–50.0% (A); 10–18 min, linear gradient 50.0–58.0% (A), 18–25 min, linear gradient 58.0–80.0% (A). A pre-balance period of 5 min was used between each run. The injection volume was 5 μL, and the flow rate was set at 0.4 mL/min. To get the fingerprints of the AR extracts, the wavelength of a ultraviolet (UV) detector was set to 254 nm with full spectral scanning from 190 to 400 nm. For the MS/MS analysis, an Agilent triple quadrupole tandem mass spectrometry (QQQ-MS/MS, 6410A) equipped with an electron spray ionization (ESI) ion source was operated in negative ion mode. The temperature and the flow of drying gas were set to 325 °C and 10 L/min, respectively. The delta electro multiplier voltage was set to 400 V, and the capillary voltage was set to 4000 V. Two transition pairs were chosen for acquisition in MRM mode for the chemical standards and the internal standard ginsenoside Rg1. The collision energy value and fragmentor voltage were optimized in advance to obtain the highest abundance.

### 4.3. Cell Culture

H9C2 cell, a cardiomyocyte cell line, was obtained from American Type Culture Collection (ATCC, Manassas, VA, USA). H9C2 cells were grown in high-glucose Dulbecco’s modified Eagle’s medium (DMEM), supplemented with 10% fetal bovine serum (FBS) and 100 units/mL penicillin/streptomycin in a humidified CO_2_ (5%) incubator at 37 °C. The culture reagents were purchased from Invitrogen Technologies (Carlsbad, CA, USA). The culture medium was replaced every 2–3 days, and the cells were grown to 80–90% confluence for experimental use.

### 4.4. Cell Viability

Cell viability was measured by the 3-(4,5-dimethylthiazol-2-yl)-2,5-diphenyltetra-zolium bromide (MTT; Sigma-Aldrich, St Louis, MO, USA) assay. The cells were seeded in 96-well plates at a density of 1 × 10^4^ cells per well. After 24 h of drug treatment, the cells in each well were incubated with 10 μL MTT (5 mg/mL, Invitrogen) at a final concentration of 0.5 mg/mL for 2 h at 37 °C. After the solution was removed, DMSO was used to re-suspend the purple precipitate inside the cells, and the absorbance was detected at 570 nm. The cell viability was calculated as percentage of the absorbance value of the control (without drug treatment), while the value of the control was 100%.

### 4.5. Folin–Ciocalteu Assay

The total phenolic content of the AR extracts was measured with the Folin–Ciocalteu assay. To be specific, 20 μL of each extract together with 40 μL 10% (*v*/*v*) of Folin–Ciocalteu reagent (Sigma-Aldrich) was added into each well of a 96-well microplate. Then, 160 μL Na_2_CO_3_ (700 mM) was added into each well. The assay plates were incubated at room temperature in the dark for 2 h, and then the absorbance at 765 nm was recorded. Here, gallic acid (Sigma-Aldrich, >98%) was used as the reference compound, and the total phenolic content of each extract was expressed in comparison to gallic acid.

### 4.6. DPPH Radical Scavenging Assay

The free radical scavenging activity of the herbal extracts was measured with the DPPH radical scavenging assay. Briefly, 50 μL of each extract at different concentrations (0–8 mg/mL) was mixed with 150 μL of DPPH solution in each well of a 96-well microplate. After standing for 10 min, the absorbance at 517 nm was recorded. The DPPH free radical scavenging activity was calculated as an inhibition percentage based on the following equation: Inhibition (%) = 100 × (A_0_ − A_1_)/A_0_, where A_0_ is the absorbance of the control, and A_1_ is the absorbance of the AR sample aliquot. Here, gallic acid (0–100 μM) was used as a positive control.

### 4.7. tBHP-Induced Oxidative Stress Assay

The doses of tBHP (150 μM; Sigma-Aldrich, St. Louis, MO, USA) and of the positive control (vitamin C, 1 mM; Sigma-Aldrich, St. Louis, MO, USA) were optimized with the MTT assay. Similar to the cell viability assay, the cells were cultured in a 96-well plate first. After drug treatment for 24 h, tBHP (150 μM) was added into the wells for 3 h, before MTT, at a final concentration of 0.5 mg/mL, was added. After the solution was removed, the purple precipitate inside the cells was resuspended in DMSO and then measured at 570 nm wavelength.

### 4.8. ROS Formation Assay

The measurement of ROS content in the cell cultures was performed by using 2′,7′-dichlorofluorescin diacetate (DCFH-DA), an oxidation-sensitive dye. Cultured H9C2 cells (1 × 10^4^ cells/well) in a 96-well plate were pre-treated with the herbal extracts or the standard compounds for 24 h, and the cells were labeled with 100 μM DCFH-DA (Sigma-Aldrich, St. Louis, MO, USA) in HBSS (Hank’s Balanced Salt Solution, Sigma-Aldrich, St. Louis, MO, USA) for 1 h at 37 °C. After washing three times with HBSS, the cells were treated with 150 μM tBHP for 1 h at 37 °C. Then, the amount of intracellular tBHP-induced ROS formation was detected by a fluorometric measurement with excitation at 485 nm and emission at 530 nm. 

### 4.9. Measurement of the Mitochondrial Membrane Potential

The mitochondrial membrane potential of tBHP-treated H9C2 cells was determined using JC-1 (5,5′,6,6′-tetrachloro-1,1′,3,3′-tetraethylbenzimidazolyl-carbocyanine iodide; Sigma-Aldrich, St. Louis, MO, USA), a fluorescent probe. H9C2 cells were cultured on a 96-well black multiwell plate with a clear bottom for 24 h. After staining with JC-1, the cells were washed twice with PBS-A and incubated with the examined compounds. The fluorescence of JC-1 aggregates in each sample was measured at an excitation wavelength of 527 nm and an emission wavelength of 590 nm every 5 min for 60 min at 37 °C. Carbonyl cyanide-p trifluoromethoxyphenylhydrazone (FCCP, 100 µM; Sigma-Aldrich, St. Louis, MO, USA), a chemical uncoupler, was used as the positive control. The data were expressed as the ratio of fluorescence intensity of JC-1 aggregates at different time points relative to the initial (i.e., 0 min) value.

### 4.10. Mitochondrial Bioenergetic Analysis

The mitochondrial bioenergetics of H9C2 cells was measured using a Seahorse Bioscience XFp extracellular flux analyzer (Agilent, Santa Clara, CA, USA), which could measure the real-time oxygen consumption by mitochondria in live cells. According to previous research, the seeding density of H9C2 cell was optimized at 5000 cells per well, and mitochondrial agents (Seahorse Bioscience Cell Mito Stress Test Kit #103010-100; North Billerica, MA, USA) were pre-optimized to elicit the maximal effects on mitochondrial respiration as follows: 1 μM oligomycin (complex V inhibitor), 3 μM FCCP (a respiratory uncoupler), and 1 μM rotenone/antimycin A (inhibitors of complex I and complex III). Background correction wells were used to normalize the data to the background noise. Cultured H9C2 cells were seeded on the XFp cell culture mini-plates and treated with the AR extracts and compounds for 24 h. After the drug treatment, the sensor cartridge of the XFp analyzer was hydrated in a non-CO_2_ incubator at 37 °C. Before sensor calibration, the cells were treated with 30 μM tBHP for 1 h and then incubated at 37 °C in a non-CO_2_ incubator in XF Base Medium (10 mM glucose, 1 mM pyruvate and 2 mM l-glutamine, pH 7.4) for another 1 h. After calibrating the sensor, the plate was placed onto the XFp extracellular flux analyzer for Mito Stress Test. The OCR was recorded over time and normalized to cellular protein content/well and corrected for extra mitochondrial O_2_ consumption. All experiments were repeated four times. Eventually, six parameters of mitochondrial function were calculated from the bioenergetics profile: basal respiration, ATP production, proton leak, maximal respiration, spare respiration capacity, and non-mitochondrial respiration.

### 4.11. Statistical Analysis

Quantitative data acquisition and processing were conducted using Agilent Mass Hunter workstation software version B.01.00 (Agilent Technologies Inc., Santa Clara, CA, USA). Principal component analysis (PCA) of the peak areas of the standard compounds was performed using SIMCA-P version 12.0 (Umetrics, Umeå, Sweden). The resultant bioenergetics profiles were analyzed with Wave Desktop 2.3.0 (Seahorse Bioscience, North Billerica, MA, USA). All data were expressed as the mean ± SEM for *n* = 3 to 5, unless otherwise specified. Statistical tests were performed by one-way ANOVA with multiple comparisons using Dunnett’s test. Differences were considered significant at *p* < 0.05.

## Figures and Tables

**Table 1:** Quantitative assessment of four chemical standards in different AR extracts.

Standards	AR-1 ^1^			AR-2			AR-3		
	W ^2^	E50	E90	W	E50	E90	W	E50	E90
Calycosin	522.3 ± 6.32 ^3^	491.56 ± 6.12	468.26 ± 2.92	467.23 ± 5.86	399.45 ± 7.42	333.22 ± 1.32	498.22 ± 5.89	387.22 ± 5.49	318.98 ± 8.19
Astragaloside IV	686.67 ± 4.23	579.23 ± 3.12	543.98 ± 2.39	537.28 ± 2.89	469.02 ± 2.19	450.22 ± 1.29	543.33 ± 5.67	582.11 ± 5.92	512.45 ± 4.89
Genistein	6.58 ± 0.13	9.14 ± 0.21	7.03 ± 0.22	5.43 ± 0.11	8.99 ± 0.32	7.93 ± 0.12	4.89 ± 0.21	5.89 ± 0.11	5.83 ± 0.12
Formononetin	139.34 ± 7.23	192.29 ± 1.91	179.34 ± 1.21	169.18 ± 5.38	187.29 ± 2.99	166.98 ± 1.11	169.29 ± 4.89	188.27 ± 3.22	138.39 ± 8.29

^1^ Three batches of AR, purchased from Shanxi province of China, were used in the present study. ^2^ W, water extracts of AR; E50, 50% ethanol extracts of AR; E90, 90% ethanol extracts of RA. ^3^ The values are expressed in mg/g of dried powder of RA, mean ± SD, *n* = 3.

## Supplementary Materials

Supplementary materials can be found at http://www.mdpi.com/1422-0067/19/6/1574/s1.
