# Supplementary material for: The Extracts and Major Compounds Derived from Astragali Radix Alter Mitochondrial Bioenergetics in Cultured Cardiomyocytes: Comparison of Various Polar Solvents and Compounds
Source: Int J Mol Sci. 2018 May 25;19(6):1574. doi: 10.3390/ijms19061574 (PMC6032251; doi:10.3390/ijms19061574)
Supplement: Supplementary file 1 [file ijms-19-01574-s001.pdf]

Supplementary data

The extracts and major compounds deriving from Astragali Radix alter mitochondrial bioenergetics in cultured cardiomyocytes: comparison of various polar solvents and compounds

Yun Huang, [1,2] Kenneth Kin Leung Kwan,[2] Ka Wing Leung,[1,2] Huaiyou Wang,[1] Xiang Peng Kong,[1] Tina Ting Xia Dong,[1,2] Karl Wah Keung Tsim[1,2,]*

[1]Shenzhen Key Laboratory of Edible and Medicinal Bioresources, Shenzhen Research Institute, Hi-Tech Park, Nanshan, Shenzhen, 518000, China; yhuangbr@connect.ust.hk (Y.H.); lkwing@ust.hk (K.W.L.); hyw@ust.hk (H.W.); xpkong@ust.hk (X.P.K.); botina@ust.hk (T.T.X.D.)

[2]Division of Life Science and Center for Chinese Medicine, The Hong Kong University of Science and Technology, Clear Water Bay, Hong Kong, China; klkwan@connect.ust.hk

*****Correspondence: botsim@ust.hk; Tel.: +852-2358-7332

List of Contents

Table S1. Mass spectra properties of marker chemicals in ginseng extracts.

Table S2. Calibration curves, LOD, LOQ of 4 major compounds in AR extracts.

Table S3. Precision, repeatability and recovery of 4 marker chemicals in AR extracts.

Figure S1. Optimization of tBHP dose in MTT assay and ROS formation assay. Cultured H9C2 cells (1×10^4 cells/well) were exposed to tBHP at various concentrations. The cell viability was determined by MTT assay after treated for 3 hours, and the level of intracellular ROS was measured by fluorescent staining after 1 hour. Cell viability was expressed as % of control (cells without tBHP), and ROS level was in % of increase (against ROS in control). tBHP at 150 μM was used for routine analysis (indicated by an arrow).

Figure S2. The effects of AR extracts and major compounds to cell viability of H9C2 cells. Cultured H9C2 cells were treated with AR extracts (0-4 mg/mL) and its compounds (astragaloside IV 0-200 μM; other compounds 0-20 μM) for 24 hours. Cell viability was determined by MTT assay. Data are expressed as mean ± SD, $n = 5$, each with triplicate samples. $*p < 0.05$.

Figure S3. Schematic representation of metabolic parameters of mitochondrial respiration measured by Seahorse Bioscience XFp extracellular flux analyzer. Basal respiration represents energetic demand of the cell under baseline conditions. Proton leak shows the remaining basal respiration and is the difference in OCR after oligomycin and rotenone/antimycin A (R&A) injection. ATP production is the difference between basal respiration and proton leak and represents the portion of basal respiration that is being used to drive ATP production. Maximal respiration shows the maximum rate of respiration that the cell can achieve, which is calculated as the OCR after FCCP injection. Spare respiratory capacity is the difference between maximal and basal OCR and can be an indicator of cell fitness or flexibility. The non-mitochondrial rate was subtracted from all other rates, which is a result of a subset of cellular enzymes that continue to consume oxygen after rotenone/antimycin A addition.

Table S1. Mass spectra properties of marker chemicals in ginseng extracts.

Chemical	Formula	Calculated mass [M]	Precursor ion [M-H][1)]	Fragmentor energy	Collison energy	Product ion[2)]
Calycosin	$C_{16}H_{12}O_5$	284.1	283.1	100	13	268
					29	211
Astragaloside IV	$C_{41}H_{68}O_{14}$	784.9	829.5[3)]	190	5	829.5
					25	783.2
Genistein	$C_{15}H_{10}O_5$	270.2	269.2	150	5	269.2
					15	225.4
Formononetin	$C_{16}H_{12}O_4$	268.1	267.1	150	17	252
					29	223
Ginsenoside Rg_1	$C_{42}H_{72}O_{24}$	800.5	799.5	250	5	799.5
					21	637.3

[1)]The detected chemicals had better responses under the negative mode: the $[M-H]^-$ was used as the precursor ion.
[2)]Two pairs of collision energy and product ions were used for the MRM analysis to guarantee the precision of analytes.
[3)]The precursor ion of astragaloside IV was $[M + HCOOH - H]^-$ under the negative mode.

Table S2. Calibration curves, LOD, LOQ of 4 major compounds in AR extracts.

Compounds	Calibration curve[1)]	Correlation coefficient (r^2)	Range (ng/mL)	LOD (ng/mL)[2)]	LOQ (ng/mL)[3)]
Calycosin	y =1764.2x - 2493.2	0.9997	1-1000	0.825	2.81
Astragaloside IV	y = 842.2x - 3102.8	0.9996	5-1000	0.536	1.85
Genistein	y = 1629.2x - 282.1	0.9993	1-1000	0.649	2.28
Formononetin	y = 2401.1x + 6109.1	0.9993	1-1000	0.677	2.21

[1)]The calibration curve was derived from six data points, *n* = 3.
[2)]LOD refers to the limits of detection.
[3)]LOQ refers to the limits of quantification.

Table S3. Precision, repeatability and recovery of 4 marker chemicals in AR extracts.

Compounds	Precision (n = 6)				Repeatability (n = 5)		Recovery[3] (n = 3)	
	Intra-day[1]		Inter-day[2]					
	Mean (ng/mL)	RSD (%)	Mean (ng/mL)	RSD (%)	Mean (ng/mL)	RSD (%)	Mean (%)	RSD (%)
Calycosin	50.2	2.38	50.8	2.93	522.3	1.21	97.6	0.65
Astragaloside IV	49.8	1.28	50.2	1.01	686.67	0.62	99.7	2.21
Genistein	48.8	2.88	49.8	2.38	6.58	1.98	97.9	2.18
Formononetin	47.9	3.29	48.2	1.29	139.34	5.19	98.1	2.21

[1)]The intra-day analysis refers to the sample examined for six replicates within one day.
[2)]The inter-day analysis refers to the sample examined in duplicates over three consecutive days.
[3)]Recovery (%) = 100 × (amount found - original amount)/amount spiked. The data were presented as average of three independent determinations.

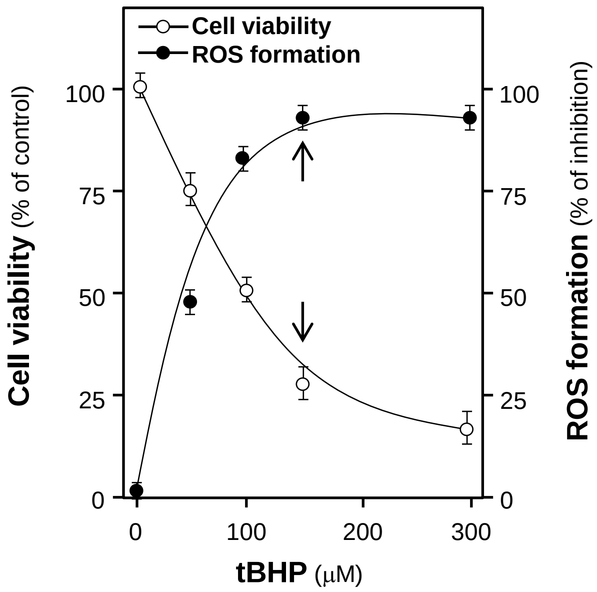

Cell viability
ROS formation
Cell viability (% of control)
ROS formation (% of inhibition)
100
75
50
25
0
0
100
200
300
tBHP (μM)

Fig. S1

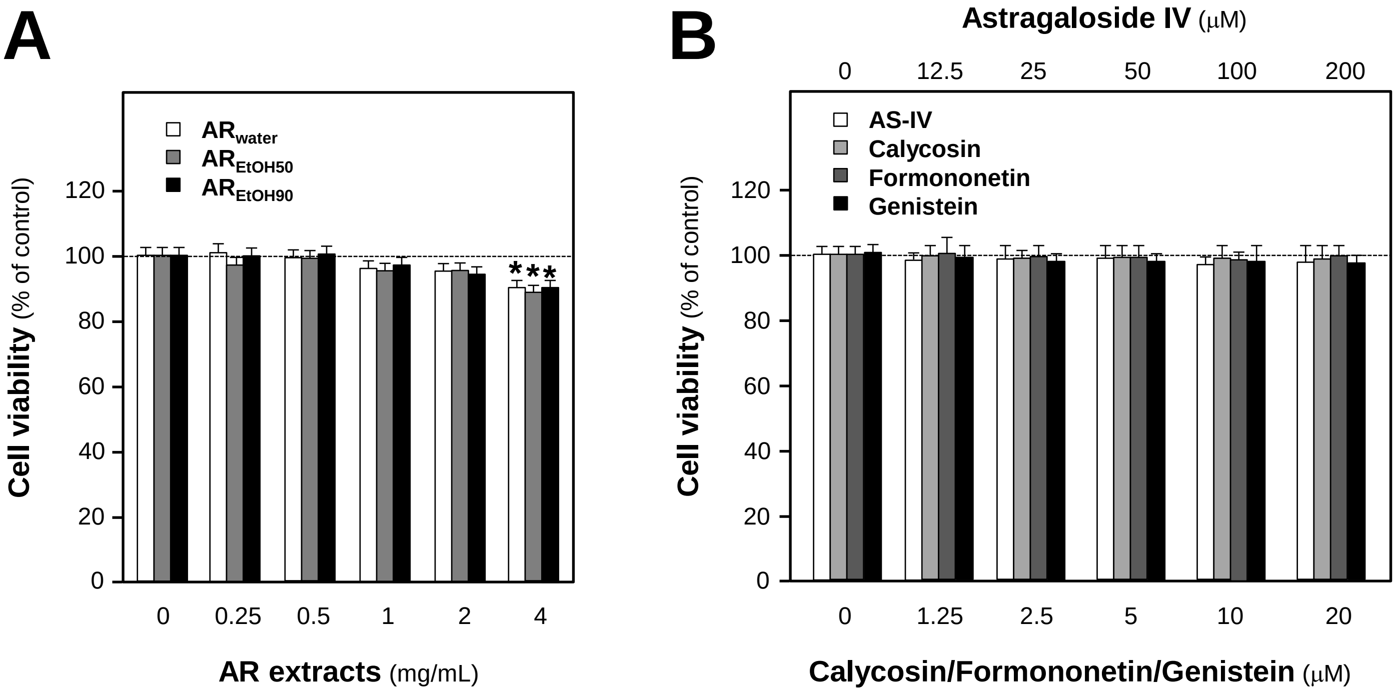
A
AR water
AR EtOH50
AR EtOH90
Cell viability (% of control)
120
100
80
60
40
20
0
0
0.25
0.5
1
2
4
AR extracts (mg/mL)
B
Astragaloside IV (μM)
0
12.5
25
50
100
200
AS-IV
Calycosin
Formononetin
Genistein
Cell viability (% of control)
120
100
80
60
40
20
0
0
1.25
2.5
5
10
20
Calycosin/Formononetin/Genistein (μM)

Fig. S2

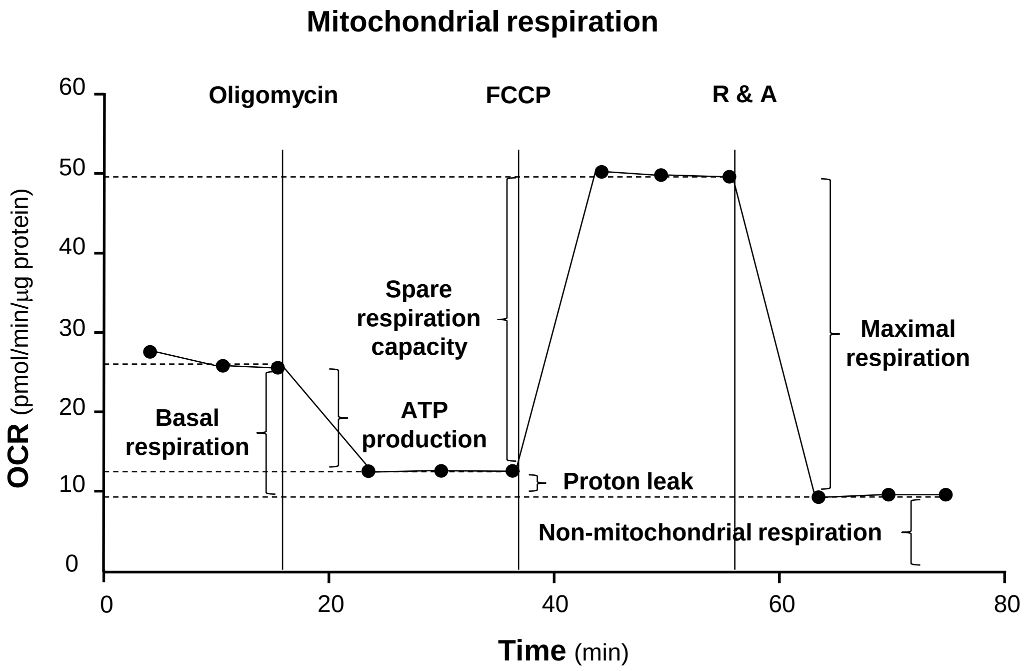
Mitochondrial respiration
Oligomycin
FCCP
R & A
OCR (pmol/min/µg protein)
60
50
40
30
20
10
0
Spare respiration capacity
Maximal respiration
Basal respiration
ATP production
Proton leak
Non-mitochondrial respiration
0
20
40
60
80
Time (min)

Fig. S3
